# The Age and Well-Being Paradox Revisited: A Multidimensional Perspective

**DOI:** 10.1093/geroni/igaa057.1485

**Published:** 2020-12-16

**Authors:** Thomas Hansen

**Affiliations:** Oslo Metropolitan University, Oslo, Norway

## Abstract

Much gerontological research has focused on the paradoxical observation that older people, despite their lower objective quality of life, report higher well-being than younger people. High well-being in old age is believed to be caused by adaptation, emotional regulation, and accommodative strategies. We aim to add nuance by examining whether the “paradox” holds across dimensions of well-being; for men and women; in young old and old-old age; and before introducing statistical controls (e.g for health and social factors). Using fixed effects models and 15-years panel data from the Norwegian NorLAG study (n=2,700, age 40+), we explore age-related changes in cognitive, affective, and eudaimonic dimensions of wellbeing. Results indicate a general pattern of stability well into older age, but negative changes in advanced age, cross-sectionally and longitudinally, and for each well-being measure. Declines in well-being are less pronounced and with a later onset for the cognitive compared with the other measures. Results are similar for men and women. Loss of health and partner are the main causes of declining well-being in older age. Findings suggest qualifications to the “well-being paradox”, e.g.: some dimensions of well-being remain more stable than others; across dimensions of well-being change is more negative in old-old than in young-old age; and patterns of increasing well-being in older age are more pronounced after controlling for age-related changes in health and social roles. We argue that the use of controls makes for false impressions of the psychological changes that actually occur when people grow older.

